# The effect of the teeth bleaching with 35% hydrogen peroxide on the tensile bond strength of metal brackets

**DOI:** 10.1038/s41598-017-00843-z

**Published:** 2017-04-11

**Authors:** Giedre Trakiniene, Simona Daukontiene, Vytautas Jurenas, Vilma Svalkauskiene, Dalia Smailiene, Kristina Lopatiene, Tomas Trakinis

**Affiliations:** 1 Lithuanian University of Health Sciences, Medical Academy, Department of Orthodontics Lithuania; 2 Lithuanian University of Health Sciences, Medical Academy, Department of Orthodontics Lithuania; 3 Associate Professor, Kaunas University of Technology Mechanical Engineering faculty, Kaunas, Lithuania; 4 Lithuanian University of Health Sciences, Medical Academy, Department of Orthodontics Lithuania; 5 Associate Professor, Lithuanian University of Health Sciences, Medical Academy, Department of Orthodontics Lithuania; 6 Associate Professor, Lithuanian University of Health Sciences, Medical Academy, Department of Orthodontics Lithuania; 7 Department of Orthopedic Surgery, Republican Hospital of Kaunas, Kaunas, Lithuania

## Abstract

The objective of this study was to determine the effects of teeth bleaching on the tensile bond strength of metal brackets bonded with light-curing adhesive system to the human enamel. 40 recently extracted human permanent molars were used for the study. The mesial buccal surface of each tooth was used as a control group and the distal buccal surface was used as an experimental group. Control group surfaces were not submitted to bleaching, while experimental group surfaces were bleached with in-office bleaching material containing 35% hydrogen peroxide. 30 days after the bleaching, identical premolar metal brackets were bonded to each surface using light-curing adhesive. Both groups were submitted to a tension test, using a universal machine. The tensile bond strength of brackets bonded to the bleached enamel was 15% lower than that of brackets bonded to the unbleached enamel. After debonding, more adhesive was left on the bracket base in experimental group than in the control group. The conclusion of this study was that bleaching with an in-office bleaching material containing 35% hydrogen peroxide reduced the tensile bond strength of orthodontic bracket adhesive to the enamel surface.

## Introduction

Standards and requirements of various services are increasing day by day and dentistry is not the exception. Nowadays, only white and aligned teeth are accepted as a beautiful smile. To fulfill these requirements, dentistry offers a number of procedures that are constantly in progress. Three decades ago, dental bleaching was quite a hazardous procedure because it could cause some side effects such as pulpal inflammation, soft tissue irritation and long lasting dental sensitivity, while today this risk still exists. The average age of orthodontic patients is rising while the average age of patients who want to bleach their teeth is decreasing. Thus it seems important to determine whether bleaching could significantly influence the bond strength of resin composites, porcelain veneers and orthodontic bracket adhesives to enamel surface^1, 2^.

There have been no reports about the effect of bleaching on the tensile bond strength measurements where both the control and the experimental groups were on the same tooth surface. In the previous studies concerning the bleaching of the teeth and the tensile bond strength, experimental teeth usually were divided into the separate groups where each tooth belonged only for one group and only one surface of the tooth was used^3, 4^. There were several studies where the same tooth was used for the control and experimental groups, but different tooth surfaces were selected, for example, labial and lingual surfaces^5^. Brosh *et al*. found that a significantly higher debonding strength was required to debond brackets from the buccal side than compared to the lingual side. Their examination with scanning electron microscope (SEM) of untreated teeth showed that pronounced horizontal ridges or perikymata run continuously around the buccal tooth surface, while in the lingual surface they appeared to a lesser degree or not at all. Moreover, their examination with SEM after acid etching procedure showed that the lingual surface had a macro-smoother pattern, smaller micropores and a less pronounced wave-like appearance after conditioning so less mechanical interlocking could develop between the resin and enamel. The authors hypothesized that the smoother appearance of the lingual surface was influenced by the continuous contact of the tongue or the presence of salivary glands^6^. Thus the estimation of teeth bleaching on the same surface and on the same tooth could be beneficial.

Studies have debated whether the differences in the adhesive remnant index (ARI) scores reflect a difference in bond strength between the enamel and the adhesive for the different adhesive systems, but adhesive systems that show less adhesive remnant on the tooth has been advocated for easier and safer removal of residual resin after debonding^7^.

The purpose of this *in vitro* study was to determine the effects of teeth bleaching on the same tooth surface on the tensile bond strength with orthodontic metal brackets.

## Materials and Methods

Approval for the study was obtained from Lithuanian University of Health Sciences Ethics Committee (No. BEC-OF-97) the methods were carried out in accordance with the relevant guidelines. The informed consent was obtained from all subjects, who participated in the study. 40 recently extracted human permanent molars (20 upper and 20 lower molars) were used for the study. The inclusion criteria for the tooth selection were as follows: intact buccal surface enamel, no caries, no restorations, no cracks from forceps, no hypoplastic areas or gross irregularities of the enamel structure. Selected teeth were examined under the 10 × 200 magnifications and from 640 × 480 to 3200 × 2400 resolution digital microscope camera (Konig Electronic CMP-USB MICRO10, The Netherlands). No pretreatment with chemical agents such as alcohol, acid or derivatives of peroxide was performed. The teeth were cleaned of blood and tissue debris, washed in the tap water and stored in the 37 °C distilled water which was changed daily to avoid bacterial growth.

The buccal surface of each tooth was divided into 2 parts: mesial buccal surfaces (N = 40) were assigned to the control (unbleached) group while all distal buccal surfaces (N = 40) were assigned to the experimental (bleached) group. As a result, 80 surfaces were used for the investigation. The mesial buccal surfaces (control group) were isolated with light curing gingival dam (FGM, Joinvile, Brazil). After that the enamel surfaces of the experimental group were bleached with 35% hydrogen peroxide (Whiteness HP Maxx, FGM, Joinville, Brazil). Bleaching agent was applied three times according to the instructions from the manufacturer. After that, the teeth were stored separately in the containers with distilled water at 37 °C for 30 days. The water was changed daily.

**Figure 1:**
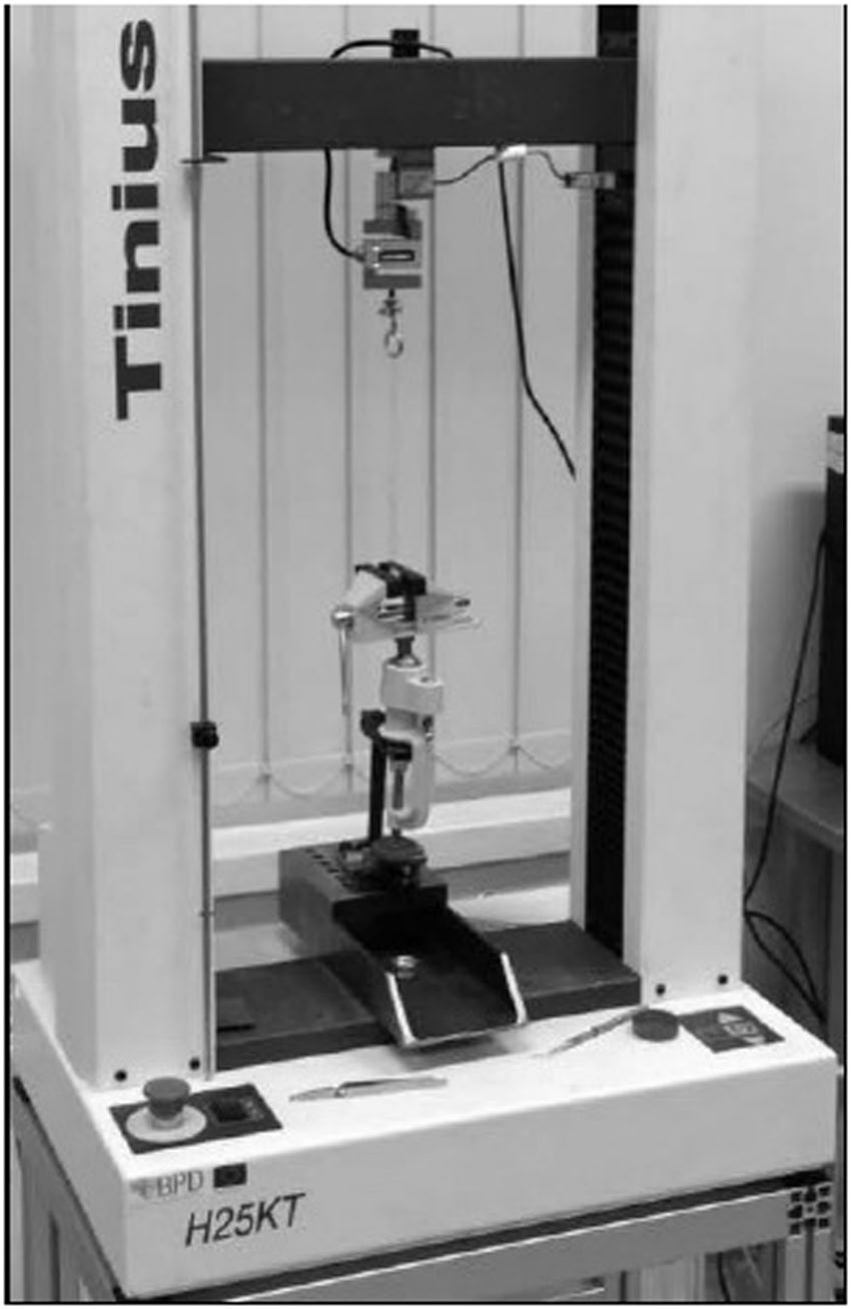
Universal testing machine (Tinius Olsen H25 KT) for the measurement of the tensile bond strength.

**Figure 2:**
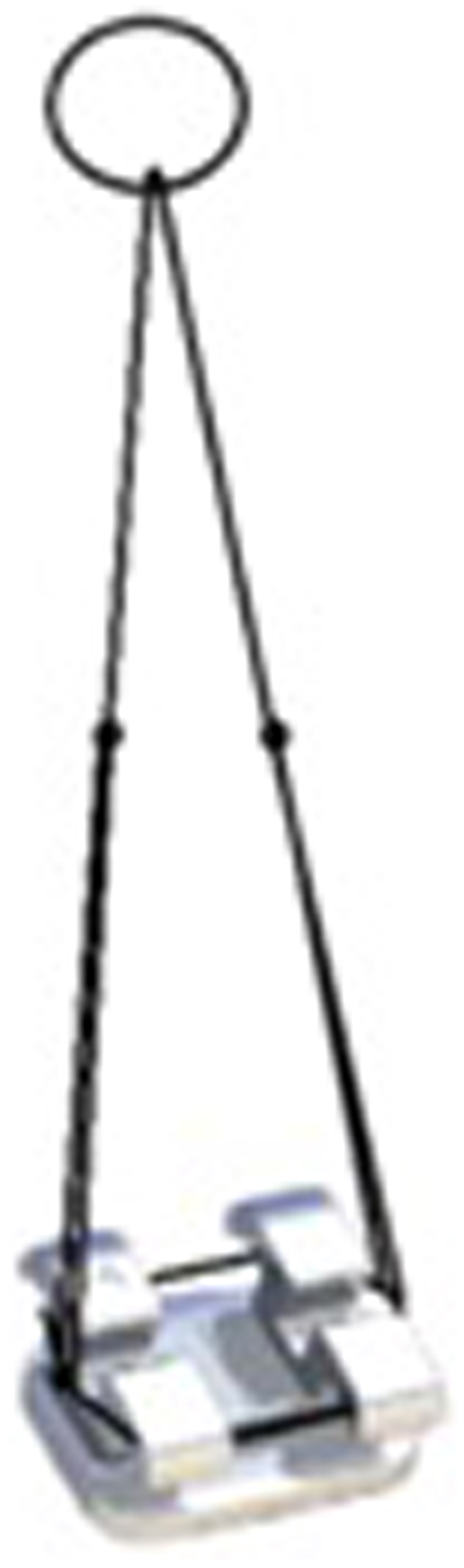
Application of the loops on the bracket.

Just before the bracket bonding, buccal surfaces of the teeth in the control and the experimental groups were cleaned and polished with rubber cup and pumice, followed by rinsing with water spray and drying with compressed air^8^. Each tooth was embedded in a vise and left there during all bonding procedure. Bonding areas were etched with 37% phosphoric acid gel for 30 seconds, then washed and dried with an oil-free compressed air for 20 seconds untill white frosty appearance was seen. Mesial buccal and distal buccal surfaces received a thin coat of the enamel bond resin, which was light-cured for 10 seconds (Translux Wave, Heraeus Kulzer, Germany, 1000 mW/cm^2^). Immediately after that, 40 identical metal brackets of upper first premolar (Dentaurum, Germany) were bonded using Transbond XT light cure adhesive paste. Each bracket was positioned on the buccal tooth surface and pressed using 100 g force through the adapter to the buccal tooth surface by the same operator for the standarisation of the thickness of the luting agent. Excess luting agent was removed with a scaler and then the resin was light-cured for 20 seconds. All the specimens were stored in the containers with distilled water at 37 °C for 1 hour until the resin was completely polymerized and then the brackets were debonded. The tensile bond strength was measured in Kaunas University of Technology Mechanical Engineering faculty laboratory. A universal mechanical testing machine (Tinius Olsen H25 KT, USA) was used at a crosshead speed of 5 mm/min with fixed loops of orthodontic wire (Remanium No. 751-001-00, Dentaurum, Germany) **(**Fig. 1). The highest forces used for debonding of the brackets were recorded automatically by a digital measurement system. The system consisted of a force sensor (SS50, Wagner instruments, USA, 250 N × 0.1 N) and a controller with a display (BGI, Wagner instruments, USA). Standardized wire loops were used for better adaptation of the sensor ring to the bracket (Fig. 2).

A digital microscope camera was used to analyze the bonded enamel surfaces and bracket bases. The photo of each bracket base was superimposed with a same size grid containing 100 cells; each of them represented 1 percent. The adhesive remnant index (ARI) was used to classify failure patterns observed in debonded specimens and was modified to a 6 point scale for achieving more exact results about bond failure localization^9, 10^. ARI scores for the debonded interfaces of the bracket base ranged as follows: 1 - no adherence of composite on the bracket base, 2 - less than 20 percent of composite remaining on the bracket surface, 3 - composite remaining on the bracket surface covers 20–40 percent of the base, 4 - composite remaining on the bracket surface covers 41–60 percent of the base, 5- composite covers 61–80 percent of the base, 6-more than 81 percent of composite remains on the bracket surface.

## Results

Data was analyzed using the SPSS 17.0 software system. The tensile bond strength data were subjected to a normality test. The data had a normal distribution, a paired t – test comparing control and experimental bond strength results for the same tooth was used to determine significance between the groups. The level of significance was established as p ≤ 0.05 with the power of the analysis 0.8.

The results showed significant reduction on the bond strength in the bleached group and (5.4 MPa; SD = 0.80) when compared to the unbleached group (6.4 MPa; SD = 1.17) and the differences were statistically significant (p = 0.0286). That means that the tensile bond strength was 15% larger in the unbleached teeth group regarding the bleached teeth group whereas the force ratio was 1.15:1.

The ARI scores were used to assess the amount of resin left on the bracket base after bracket debonding and were estimated by two trained orthodontists separately and blindly. Examiners were calibrated for inter-examiner reliability by means of Kappa statistics. The kappa values for interobserver agreement for ARI scores was 0.83 and showed almost perfect agreement. Examination of the teeth surfaces and bracket bases showed that in the control group, bracket failures mainly occurred between the bracket bases and bonding material, therefore more adhesive was left on the enamel surface compared to the bracket base. However, in experimental group, more adhesive was left on the bracket base than on the tooth surface, showing bracket failure between bleached enamel surfaces and bonding material.

The Kruskal-Wallis test (X^2^ = 17.458) indicated a statistically significant difference between ARI of bleached and unbleached groups (p ≤ 0.05). The scores of ARI varied between the two groups and inside each group: from 1 to 3 in the unbleached group and from 2 to 5 in the bleached group (Table 1).

**Table 1:** The distribution and frequency of the adhesive remnant index (ARI) scores in bleached and unbleached group (p ≤ 0.05).

Group	ARI score											
	1		2		3		4		5		6	
Unbleached	2 (5.0%)	p = 0.0156	12 (30.0%)	p = 0.0043	24(60.0%)	p = 0.0265	2 (5.0%)	p = 0.0072	0 (0.0%)	p = 0.0391	0 (0.0%)	p = 0.0156
Bleached	0 (0.0%)		0 (0.0%)		10 (25.0%)		22(55.0%)		6(15.0%)		2 (5.0%)	

## Discussion

The hypothesis that bleached enamel showed lower tensile bond strength properties than unbleached enamel was confirmed. Although various bleaching systems and techniques are currently used for teeth bleaching, there is no general consensus which bleaching technique is the best. While analyzing the effect of bond strength one should pay attention to the factors which can influence the results. These include: pre-experimental storage of the teeth, bleaching material, affected teeth surface, post bleaching period and bonding pattern.

One of the factors that may affect the bond strength is the pre-experimental storage medium. Storing teeth in the media decreases bacterial, viral, and fungal growth, prevents enamel desiccation and allows teeth to be stored before testing. Titley *et al*. investigated the effect of 11 storage media on shear bond strengths of composite resin restorations applied to bovine dentin and found that specimens which were stored in thymol, methanol, and glutaraldehyde showed lower shear bond strengths to bovine dentin compared with the controls stored in the water^11^. Another study made by Jaffer *et al*. showed that storage of enamel in the dry environment or in the ethanol solution had a negative effect on bond strength, possibly due to the desiccating effects of these storage variables^12–15^. In the recent study, the specimens before bleaching and after that were stored in 37 °C distilled water to simulate oral environment as it was done in the study of Andradae^16^.

As the variety of bleaching materials is increasing every day, in the literature we found the hydrogen peroxide to be the most popular for experimental designs. Oztas *et al*. in their study used 20% hydrogen peroxide and found no significant differences in shear bond strength in bleached and unbleached teeth groups^17^. This difference from our results could be due to the lower concentration of hydrogen peroxide used for the enamel bleaching and regarding different teeth which were used in the control and the experimental groups.

The other factor which could affect the tensile bond strength is post bleaching period. In the previous studies, this period varied from 24 hours to 4 weeks and the results were controversial. Rego *et al*. in their study found a statistically significant reduction in the bond strength when compared control group to the group where bonding was performed 24 hours after the bleaching. Furthermore, they noticed that there were no statistically significant differences between bracket debonding strength seven days after the bleaching^4^. The same findings were established in the studies conducted by Bishara and Bulut, which showed that if bonding procedure on the bleached teeth was delayed for 1 week the bond strength will increase^18–20^. However, Akin *et al*. in their study found a significant reduction in the shear bond strength of the bleached group compared with the control group when bracket bonding was performed 3 weeks after bleaching with 38% hydrogen peroxide^3^. These results coincide with our findings although different methods of bond strength measurement were used.

In addition, according to the debonding of brackets, where are two positions: manual debonding and mechanical debonding. Some authors in their studies used manual debonding in order to simulate more closely forces applied in the actual clinical situations. But by using this method, it is impossible to measure the magnitude of actual force^21, 22^. Nowadays for experimental designs which need to measure debonding force many authors use mechanical testing as we did. For that reason a lot of debonding instruments were established with different crosshead speed, but still they are not standardized^23^. However, it may be concluded that manual debonding requires considerably lower forces than traditional debonding tests and more studies are required for the standardization of these methods.

The reduction of bond strength can be explained by the effects of teeth bleaching on the organic (protein) content of the tooth and changes in the mineral phase, resulting in the morphological changes to the tooth surface^23, 24^. Shannon *et al*., in their *in-vivo* study on bleached teeth with 10% carbamide peroxide noticed that the microhardness of bleached teeth in natural saliva showed a significant increase in 4 weeks^25^. On the other hand, many authors suggest the use of remineralizing agents for at least 2 weeks for reversion of this mineral loss by the remineralization treatments with a fluoride based products or with the use of toothpastes containing bioactive glass particles, since they are able to replenish the Ca and P content of damaged enamel and return it to that of undamaged enamel^26^. The other mechanism for this is the stabilization of calcium phosphates on the tooth surface by the casein phosphopeptides, which leads to high concentration gradients of calcium and phosphate ions, thus promoting the remineralization of hard tissues^27^. Of note, further investigations should be undertaken in order to evaluate the long-term effect of these products on enamel remineralization, and their implication on enamel bonding.

Finally, talking about ARI scores, the results of recent study support the study of Uysal *et al*. The authors found significant differences in ARI scores between control and experimental groups. The unbleached group's failures were primarily at the bracket-adhesive interface, whereas the bleached groups either showed cohesive failures within the adhesive or failed at the adhesive-enamel interface^8^. This could be clinically advantageous, since, when brackets fail at the enamel-adhesive interface, less adhesive remains, and tooth cleanup is likely to be easier and faster^28, 29^. However, Reynolds has suggested that a minimum tensile bonding strength of 5.9 MPa to 8 MPa between orthodontic brackets and teeth would be adequate for clinical orthodontic tooth movement^30^. In this study the tensile bond strength in the unbleached group was 6.4 MPa and 5.4 MPa in the bleached group, thus, the bond strength in the bleached teeth group is in the borderline and may have negative effect in the producing of teeth movements.

## Limitations Of The Study

It was experimental study, thus it was impossible to repeat the tension test with the same teeth.

## Conclusions

Within the limitations of this study, we can conclude, that the use of 35% hydrogen peroxide for the in-office bleaching significantly reduced the tensile bond strength of the bonded brackets 30 days after the bleaching. After the bracket debonding from the bleached teeth more adhesive was left on the bracket base compared with the cases of the unbleached teeth.

Further investigations are needed for standardization of the measurement methods and establishment of the period when the tooth bleaching would have no influence on the bond strength values.
